# Metal-Free Synthesis of Hydrophobic and Dielectric Poly(propylene carbonate) via CO_2_/PO/TF-PO Terpolymerization: Characterization and DFT Mechanistic Analysis

**DOI:** 10.3390/polym18091057

**Published:** 2026-04-27

**Authors:** Gehui Liu, Wenzhen Wang, Bin Cao, Xinyi Liu, Xingang Jia, Leilei Li, Yefei Nan

**Affiliations:** 1 College of Chemistry and Chemical Engineering, Xi’an Shiyou University, Xi’an 710065, China; 23212071067@stumail.xsyu.edu.cn (G.L.); caobinxys@126.com (B.C.); liuxinyi80233208@163.com (X.L.); jiaxingang76@xsyu.edu.cn (X.J.); lll@xsyu.edu.cn (L.L.); nanyf@xsyu.edu.cn (Y.N.); 2 Shaanxi Engineering Research Center of Green Low-Carbon Energy Materials and Processes, Xi’an Shiyou University, Xi’an 710065, China

**Keywords:** poly(propylene carbonate), terpolymerization, hydrophobicity, dielectric properties, density functional theory

## Abstract

To overcome the inherent drawbacks of poly(propylene carbonate) (PPC), such as poor thermal stability, low mechanical strength, and high surface energy, this study introduced, for the first time, 1,1,1-trifluoro-2,3-epoxypropane (TF-PO) as a third monomer into the metal-free TEB/PPNCl catalytic system for the terpolymerization with carbon dioxide (CO_2_) and propylene oxide (PO), successfully synthesizing a series of fluorinated PPC (PPCF). The optimal polymerization conditions (60 °C, 2.0 MPa, 12 h, n(PO):n(TF-PO) = 100:4) were determined through systematic optimization. Comprehensive structural characterization (FT-IR, NMR, XPS) confirmed the successful incorporation of TF-PO into the polymer backbone. Property evaluation revealed that the PPCF materials exhibited substantial improvements in thermal stability, mechanical strength, hydrophobicity, and dielectric properties compared to unmodified PPC. The optimal sample, PPCF4, achieved a 5% weight-loss temperature ($T_{d,-5\%}$) of 242 °C, a glass transition temperature ($T_{g}$) of 42 °C, a tensile strength of 21.5 MPa, and a Young modulus of 296 MPa. With a 5% TF-PO feed ratio, the material’s water contact angle increased to 102°, and its dielectric constant reached 6.01 at $10^{4}$ Hz. Furthermore, density functional theory (DFT) calculations elucidated the Lewis acidity of the TEB catalyst and the reactive sites of the monomers, leading to a proposed mechanism for the ternary alternating copolymerization. This work provides an effective synthetic strategy and theoretical foundation for preparing high-performance and functionalized PPC materials through molecular structure design.

## 1. Introduction

The escalating global climate change and plastic pollution [1,2,3,4,5] have spurred research into synthesizing biodegradable polymers using carbon dioxide (CO_2_) as a feedstock [6,7]. Among these, poly(propylene carbonate) (PPC), produced from the copolymerization of CO_2_ and propylene oxide (PO), possesses a “carbon-negative” characteristic and excellent biodegradability, making it a promising green plastic [8,9,10,11]. The conversion of greenhouse gas CO_2_ into value-added PPC contributes to a circular carbon economy [12]. However, practical applications of PPC are limited by its poor thermal stability, low mechanical strength, and high surface energy [13,14,15], with a low glass transition temperature ($T_{g}\approx 20$–40 °C) that further restricts its utility [16,17].

Chemical modification via terpolymerization is an effective strategy to overcome these limitations. Introducing a third monomer into the CO_2_/PO system enables precise regulation of polymer architecture, synergistically improving thermodynamic properties and imparting specific functionalities [18,19,20,21,22,23,24]. For example, terpolymerization with cyclic anhydrides such as $4,4^{\prime }$-oxydiphthalic anhydride (ODPA) significantly enhances molecular weight and thermal stability through branching and end-group capping [25]. Complementary physical approaches—including reinforcement with bio-based fibers [17], incorporation of nanofillers [16], addition of renewable additives like wool powder [13], and use of eco-friendly plasticizers [14]—have also been shown to improve the mechanical, thermal, and barrier properties of PPC-based materials. Furthermore, PPC can serve as a matrix in multicomponent composites where tailored interfacial interactions yield synergistic reinforcement and toughening [26].

Fluorinated monomers, owing to the high bond energy and low polarizability of C–F bonds, can markedly reduce surface energy, conferring hydrophobicity, chemical stability, and dielectric properties [25,27]. 1,1,1-Trifluoro-2,3-epoxypropane (TF-PO), as a fluorinated epoxide, contains an epoxide ring for copolymerization and a pendant trifluoromethyl (-CF_3_) group that enhances surface and dielectric characteristics [28,29].

In the context of heterogeneous CO_2_ cycloaddition catalysts, a wide array of solid materials has been developed, including zeolitic imidazolate frameworks (ZIFs) [30], metal–organic frameworks (MOFs) [31,32,33], and porous organic polymers [34,35]. Functionalized silicas, carbon nitrides, and magnetic core–shell composites have also been explored to leverage synergies between Lewis acid sites and nucleophilic halides [36,37,38]. Despite the high activity of many metal-based frameworks (e.g., Zn, Co, Cr, or rare-earth elements) [39,40,41,42], they often suffer from metal leaching, cytotoxicity concerns, and complex syntheses [43].

Metal-free organocatalytic systems have therefore attracted increasing attention as a means to circumvent the drawbacks associated with metal residues. In particular, the discovery that triethylborane (TEB), a commercially available mild Lewis acid, can form highly active ate complexes with onium salts or organic bases has revolutionized the field of metal-free CO_2_/epoxide copolymerization [44]. This approach operates via an anionic copolymerization mechanism, wherein TEB activates the epoxide monomer while simultaneously stabilizing the propagating alkoxide chain end through B–O interactions, effectively suppressing side reactions such as cyclic carbonate formation and polyether linkages [44]. The versatility of these borane-based Lewis pairs has enabled not only the synthesis of perfectly alternating polycarbonates with high selectivity under mild conditions, but also the construction of diverse macromolecular architectures including block copolymers and telechelic polyols [45]. Despite these advances, metal-free systems often require relatively high catalyst loadings or elevated pressures to achieve satisfactory yields [46,47], underscoring the need for further optimization.

In this study, we employ the TEB/PPNCl catalyst system and, for the first time, introduce TF-PO into the CO_2_/PO copolymerization to synthesize fluorinated PPC (PPCF) with tailored hydrophobicity and dielectric performance. Polymerization conditions were systematically optimized, and the effects of TF-PO on chain structure, thermal and mechanical properties, surface wettability, and dielectric behavior were comprehensively characterized. Density functional theory (DFT) calculations were further performed to elucidate the reaction mechanism: the electrostatic potential distribution and LUMO localization of TEB confirmed its Lewis acidic boron center; frontier molecular orbital analysis revealed that TF-PO possesses a lower LUMO energy, narrower HOMO–LUMO gap, and larger dipole moment than PO, accounting for its higher reactivity in the terpolymerization.

## 2. Materials and Methods

### 2.1. Experimental Reagents and Instruments

Propylene oxide (PO), 1,1,1-trifluoro-2,3-epoxypropane (TF-PO), bis(triphenylphosphine)iminium chloride (PPNCl), and dichloromethane were of analytical grade and purchased from Shanghai Macklin Biochemical Technology Co., Ltd. (Shanghai, China), and were used as received without further purification. Triethylborane (TEB, 1.0 M solution in tetrahydrofuran) was obtained from Aladdin Reagent Company(Shanghai, China). High-purity carbon dioxide (≥99.99%) was supplied by Shaanxi Tenglong Gas Factory (Yulin, China) and used as received.

The primary instruments employed for polymer characterization were as follows. ^1^H nuclear magnetic resonance (NMR) spectra were recorded at room temperature on a Bruker BioSpin AG, Fällanden, Switzerland, AVANCE NEO 400 MHz spectrometer using CDCl_3_ as the solvent and tetramethylsilane (TMS) as the internal standard. Fourier-transform infrared (FT-IR) spectra were acquired on a Bruker BioSpin AG, Fällanden, Switzerland, VERTEX 70 spectrometer. X-ray photoelectron spectroscopy (XPS) was performed on a Thermo Fisher Scientific, Waltham, MA, USA, Thermo Scientific K-Alpha instrument. Thermogravimetric analysis (TGA) was conducted using a METTLER TOLEDO, Greifensee, Switzerland, TGA-DSC 1 analyzer. Differential scanning calorimetry (DSC) curves were recorded on a METTLER TOLEDO, Greifensee, Switzerland, DSC 822e instrument. The number-average molecular weight (${\bar{M}}_{n}$) and polydispersity index (PDI) of the polymers were determined by gel permeation chromatography (GPC) on an Agilent Technologies, Santa Clara, CA, USA, PL-GPC 50 system equipped with a refractive index detector, using tetrahydrofuran (THF) as the eluent at a flow rate of 1.0 mL/min and calibrated with narrow-polydispersity polystyrene standards. Mechanical properties were measured with an electronic universal testing machine (MTS Systems (China) Co., Ltd., Shenzhen, China, CMT-6104). For cytotoxicity assays, L929 fibroblast cells were cultured overnight in a humidified incubator at 37 °C under a 5% CO_2_ atmosphere. The cells were treated with polymer solutions at five gradient concentrations prepared in DMSO. After 24 h of incubation, 100 μL of CCK-8 solution was added to each well, followed by an additional 4 h of incubation, and the absorbance was measured at 450 nm using a microplate reader. A high-pressure autoclave (YBES-100ML, Aike Experimental Instrument Co., Ltd., Xi’an, China) equipped with magnetic stirring and electric heating was used for all polymerization reactions.

### 2.2. Synthesis of PPCF

**Figure 1:**
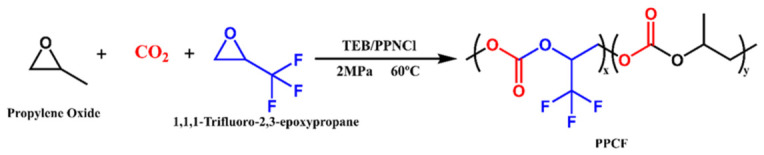
Ternary copolymerization of CO_2_, PO, and TF-PO.

All polymerization reactions (Figure 1) were conducted in a 100 mL high-pressure autoclave equipped with magnetic stirring and electric heating capabilities. The specific synthesis procedure was as follows: First, bis(triphenylphosphine)iminium chloride (PPNCl, 0.287 g, 0.5 mmol) was accurately weighed and placed into the autoclave. Subsequently, a fixed amount of PO (5.8 g, 100 mmol) and TF-PO monomer were added sequentially according to the predetermined molar ratios [n(PO):n(TF-PO) = 100:1 to 100:5]. Then, triethylborane (TEB, 1 mL, 1 mmol) was introduced. After sealing the autoclave, CO_2_ gas was charged to adjust the internal pressure to 2 MPa. The reaction system was maintained at 60 °C for 12 h with constant stirring. Upon completion of the reaction, the mixture was quenched with 5 mL of a 5% hydrochloric acid–ethanol solution. The resulting crude product was dissolved in 20 mL of dichloromethane, then precipitated in 90 mL of anhydrous ethanol. The precipitated product was collected and finally dried to a constant weight in a vacuum oven at 60 °C, yielding the final PPCF product.

### 2.3. Theoretical Calculation Methods

All quantum chemical calculations were performed using the Gaussian 16 program package [48] under the framework of density functional theory (DFT). Geometry optimizations and vibrational frequency analyses were carried out at the B3LYP/6-31G(d) level of theory. For structurally complex molecular assemblies, initial geometries were pre-optimized at the PM6 semi-empirical level prior to full DFT treatment. All stationary points were rigorously characterized by harmonic vibrational frequency calculations: equilibrium structures exhibited no imaginary frequencies, whereas transition states possessed exactly one imaginary frequency corresponding to the reaction coordinate.

Based on the optimized geometries, a comprehensive analysis of the reaction mechanism was undertaken from several complementary perspectives:

Frontier Molecular Orbital Analysis: The energies and spatial distributions of the highest occupied molecular orbital (HOMO) and the lowest unoccupied molecular orbital (LUMO) of the reactants were evaluated to assess their relative reactivity and to identify the dominant orbital interaction patterns governing the polymerization process.

Geometric Structure Analysis: The evolution of key structural parameters, including bond lengths, bond angles, and dihedral angles, was monitored along the reaction pathways to elucidate the structural reorganization accompanying bond formation and cleavage.

Charge Distribution Analysis: Electrostatic potential maps and atomic partial charges were computed to characterize the charge distribution within key intermediates, providing a complementary perspective to the frontier orbital analysis and helping to rationalize the regioselectivity of nucleophilic attacks.

To corroborate the structural assignments derived from experimental ^1^H NMR spectroscopy, the magnetic shielding tensors and spin–spin coupling constants for a representative fluorinated poly(propylene carbonate) (PPCF) segment were computed using the gauge-including atomic orbital (GIAO) method. The NMR calculations were performed at the B3LYP/6-31G(d) level of theory, incorporating the solvent effects of chloroform via the integral equation formalism variant of the polarizable continuum model (IEFPCM).

**Figure 2:**
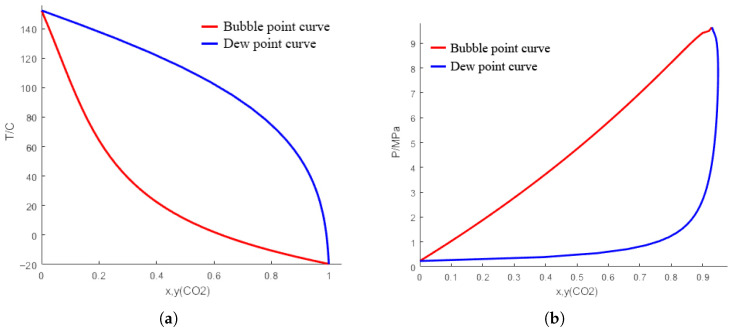
Vapor–liquid equilibrium phase diagrams for the carbon dioxide–propylene oxide system: (**a**) *T*–xy diagram at a pressure of 2 MPa; (**b**) *P*–xy diagram at a temperature of 60 °C.

In addition to the quantum chemical calculations described above, the vapor–liquid equilibrium (VLE) behavior of the CO_2_/propylene oxide binary system was modeled using the Soave–Redlich–Kwong (SRK) equation of state. A custom C++ program was developed to perform bubble-point and dew-point calculations, and the resulting *T*–xy and *P*–xy phase diagrams are presented in Figure 2. The binary interaction parameter $k_{ij}$ employed in the SRK mixing rules was taken from the literature [49,50], and the pure-component critical properties and acentric factors were obtained from the NIST Chemistry WebBook database [51]. Table 1 and Table 2 summarize the thermodynamic parameters used in these calculations.

## 3. Results

To obtain PPCF products with high molecular weight and yield, this study systematically investigated the effects of reaction temperature, pressure, and time on the polymerization. As summarized in Table 3, Table 4 and Table 5, the polymerization outcome exhibited a marked dependence on these operational parameters. When the temperature was raised from 40 °C to 60 °C, the yield increased from 17% to 78%, the number-average molecular weight (${\bar{M}}_{n}$) rose from 4.21 kDa to 20.67 kDa, and the polydispersity index (PDI) narrowed from 2.43 to 1.18. At 70 °C, the yield decreased to 71%, ${\bar{M}}_{n}$ dropped to 11.45 kDa, and the PDI broadened to 1.49. With respect to pressure, the maximum yield (78%), the highest ${\bar{M}}_{n}$ (20.67 kDa), and the narrowest PDI (1.18) were attained at 2.0 MPa, whereas both lower and higher pressures resulted in inferior outcomes. Prolonging the reaction time from 12 h to 16 h yielded no appreciable improvement in yield or ${\bar{M}}_{n}$, indicating that the polymerization was essentially complete within 12 h. On the basis of these observations, the optimal polymerization conditions were identified as a temperature of 60 °C, a CO_2_ pressure of 2.0 MPa, and a reaction time of 12 h.

The dependence of the copolymerization performance on temperature and CO_2_ pressure (Table 3, Table 4 and Table 5) can be interpreted in light of the vapor–liquid equilibrium (VLE) behavior of the CO_2_/propylene oxide binary system (Figure 2) and the established mechanistic understanding of CO_2_/epoxide copolymerization. At a constant pressure of 2 MPa, the *T*–xy diagram (Figure 2a) shows that the reaction mixture resides in the two-phase region across the temperature range investigated. At the optimal temperature of 60 °C, the liquid phase contains a substantial mole fraction of dissolved CO_2_ ($x_{{\mathrm{CO}}_{2}}\approx 0.30$), ensuring a high concentration of CO_2_ available for insertion into the growing polymer chain. Elevating the temperature to 70 °C shifts the operating point closer to the dew-point boundary, reducing the equilibrium CO_2_ solubility in the liquid phase. This decrease in dissolved CO_2_ concentration favors competitive side reactions, such as epoxide homopolymerization or chain transfer, over alternating copolymerization, which is consistent with the observed decline in ${\bar{M}}_{n}$ and broadening of PDI at 70 °C (Table 3) [52].

Regarding the pressure effect, the *P*–xy diagram at 60 °C (Figure 2b) reveals that at lower pressures (e.g., 1 MPa), the liquid-phase CO_2_ concentration is significantly reduced ($x_{{\mathrm{CO}}_{2}}<0.20$), leading to insufficient CO_2_ incorporation and the formation of polyether-rich copolymers [52], resulting in low yields and molecular weights (Table 4). As the pressure is increased to 2 MPa, the dissolved CO_2_ concentration in the liquid phase rises markedly, thereby facilitating the alternating insertion of CO_2_ and propylene oxide. Kinetic studies on related heterogeneous catalytic systems have demonstrated that the rate of CO_2_/epoxide copolymerization is zero-order with respect to CO_2_ pressure once the liquid phase is saturated with CO_2_ [52]. This implies that further increasing the pressure beyond the saturation threshold does not enhance the intrinsic reaction rate but may instead dilute the epoxide concentration in the liquid phase [49]. Such dilution can reduce the frequency of epoxide coordination to the active sites, which is the rate-determining step, thereby accounting for the slight decrease in yield and ${\bar{M}}_{n}$ observed at 3 MPa (Table 4). Notably, the concept of a “CO_2_-expanded” liquid phase, where the system operates in the two-phase region near the mixture’s critical point, has been identified as the optimal regime for maximizing catalytic productivity in CO_2_/epoxide copolymerizations [49]. The conditions of 60 °C and 2.0 MPa place the reaction precisely within this advantageous CO_2_-expanded regime, striking an optimal balance between high CO_2_ availability in the liquid phase and favorable epoxide coordination kinetics.

Table 6 presents the copolymerization results of PO and TF-PO at different molar ratios. The TEB system effectively catalyzed the ternary copolymerization of CO_2_, PO, and TF-PO. With increasing TF-PO content, the yield initially improved. The introduction of a small amount of TF-PO led to competitive insertion between CO_2_ and TF-PO, disrupting the binary copolymerization equilibrium and promoting the reaction progress, thereby simultaneously enhancing both the product yield and molecular weight (${\bar{M}}_{n}$). However, when the molar ratio of PO to TF-PO reached 100:5, the relative concentration of PO decreased, resulting in a decline in both yield and ${\bar{M}}_{n}$. In summary, the optimal feed ratio was determined to be 100:4.

### 3.1. Confirmation of the Chemical Structure of PPCF

**Figure 3:**
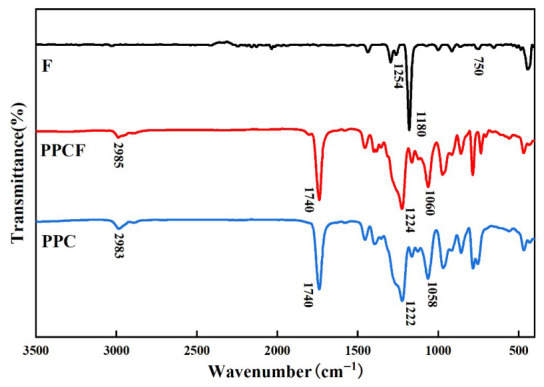
FT-IR spectra of 1,1,1-trifluoro-2,3-epoxypropane (TF-PO), PPCF, and PPC.

The polymer structure was characterized using Fourier transform infrared spectroscopy (FT-IR) (Figure 3). PPCF exhibited a characteristic peak at $1740\;{\mathrm{cm}}^{-1}$ corresponding to the ester C=O group, while peaks at $1224\;{\mathrm{cm}}^{-1}$ and $1060\;{\mathrm{cm}}^{-1}$ were assigned to the ester C-O and ether C-O-C linkages, respectively, indicating successful polymerization between CO_2_ and the epoxides. The stretching vibration peaks of the -CH_3_ group at $2980\;{\mathrm{cm}}^{-1}$ and $2890\;{\mathrm{cm}}^{-1}$ were consistent with those of PPC. Compared to TF-PO, the characteristic epoxy ring peaks at $1254\;{\mathrm{cm}}^{-1}$ and $750\;{\mathrm{cm}}^{-1}$ disappeared in PPCF, confirming the ring-opening of the epoxide groups. Due to steric hindrance effects, the absorption peak of the C-F bond might not be distinct or could appear shifted.

**Figure 4:**
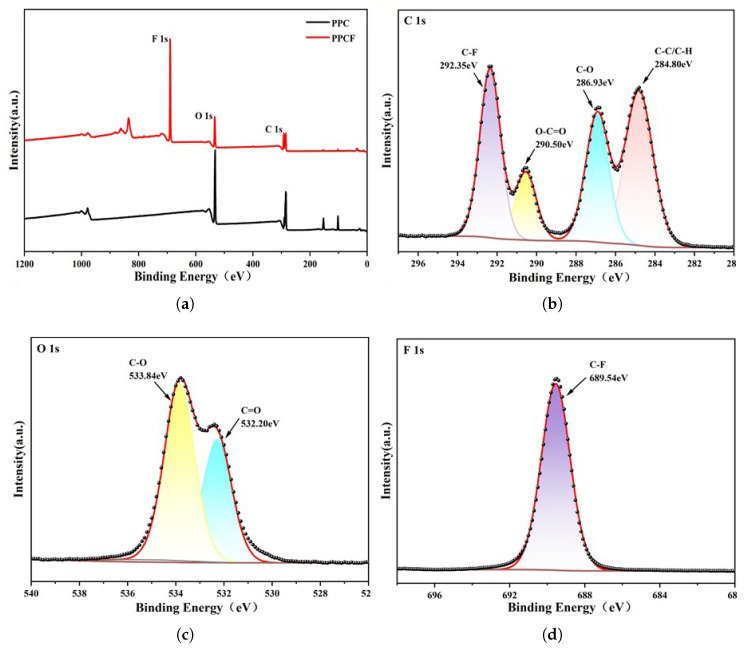
(**a**) XPS survey spectra of PPC and PPCF; (**b**) high-resolution C 1s spectrum; (**c**) high-resolution O 1s spectrum; (**d**) high-resolution F 1s spectrum.

The surface chemical composition and bonding states of the synthesized PPCF copolymer were further elucidated by X-ray photoelectron spectroscopy (XPS). The survey spectrum (Figure 4a) confirmed the presence of fluorine in PPCF alongside carbon and oxygen, whereas only C and O were detected in the unmodified PPC. High-resolution core-level spectra provided detailed information on the local chemical environments. The C 1s spectrum (Figure 4b) was deconvoluted into four components: the dominant peak at 284.8 eV is assigned to aliphatic C-C/C-H bonds, while the peaks at 286.5 eV, 289.0 eV, and 292.2 eV correspond to C-O, O-C=O (carbonate), and C-F (trifluoromethyl) groups, respectively. The O 1s spectrum (Figure 4c) exhibited two distinct peaks at 532.2 eV and 533.8 eV, attributed to carbonyl (C=O) and ether-type (C-O) oxygen atoms within the carbonate linkages. The F 1s spectrum (Figure 4d) displayed a single symmetric peak at 689.3 eV with a full width at half maximum (FWHM) of 1.73 eV, characteristic of covalently bonded fluorine in -CF_3_ moieties.

Quantitative elemental analysis derived from the XPS survey data is summarized in Table 7. Notably, the surface fluorine content reached 36.88 at. %, corresponding to an atomic F/C ratio of approximately 0.80. This value significantly exceeds the bulk stoichiometric ratio anticipated from the TF-PO feed, indicating a pronounced surface enrichment of the fluorinated segments. Such segregation is thermodynamically driven by the low surface energy of the -CF_3_ groups and has profound implications for the macroscopic properties of the material: (i) the accumulation of hydrophobic -CF_3_ moieties at the air–polymer interface is responsible for the enhanced water contact angles and reduced water vapor transmission; (ii) the highly polarized C-F bonds contribute to the increased dielectric constant through enhanced orientational polarizability; and (iii) the strong electron-withdrawing nature of fluorine reinforces interchain dipole–dipole interactions, which is consistent with the observed elevation in glass transition temperature and mechanical stiffness. The XPS results thus not only confirm the successful covalent incorporation of TF-PO into the PPC backbone but also provide a mechanistic rationale for the comprehensive improvements in thermal, mechanical, surface, and dielectric properties described in the subsequent sections.

**Figure 5:**
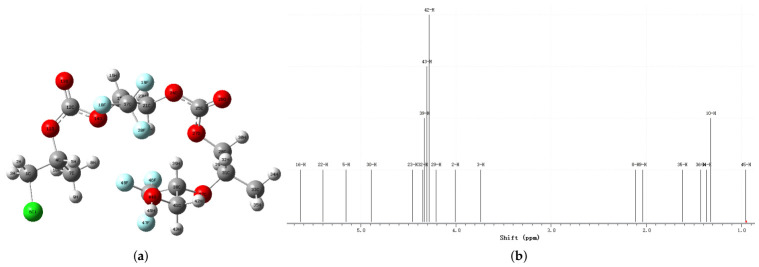
(**a**) DFT-optimized geometry of a representative PPCF segment. (**b**) Simulated ^1^H NMR spectrum derived from the optimized structure.

To further verify the successful incorporation of 1,1,1-trifluoro-2,3-epoxypropane (TF-PO) into the PPC chains and to elucidate the polymer microstructure, the chemical structure was analyzed by ^1^H NMR spectroscopy complemented by DFT calculations. Geometry optimization and subsequent GIAO (Gauge-Including Atomic Orbital) NMR chemical shift prediction were performed on a representative PPCF segment using Gaussian 16 at the B3LYP/6-31G(d) level with chloroform solvation (Figure 5). The DFT-computed chemical shifts for the methine and methylene protons of the TF-PO and PO repeat units fall within a closely overlapping range, indicating that the intrinsic electronic environments of these protons in the two monomeric units are remarkably similar. Furthermore, the conformational heterogeneity of the flexible polymer chain in solution induces a distribution of local magnetic environments, leading to inhomogeneous broadening of the NMR signals. As a consequence of the near-degeneracy of the calculated chemical shifts combined with conformational averaging, the resonances of the PO and TF-PO backbone protons coalesce into a single, broad signal envelope in the experimental spectrum, which is fully consistent with the observed feature between 4.1 and 4.3 ppm. The simulated spectrum exhibits good overall agreement with the experimental data, providing support for the proposed spectral assignments.

**Figure 6:**
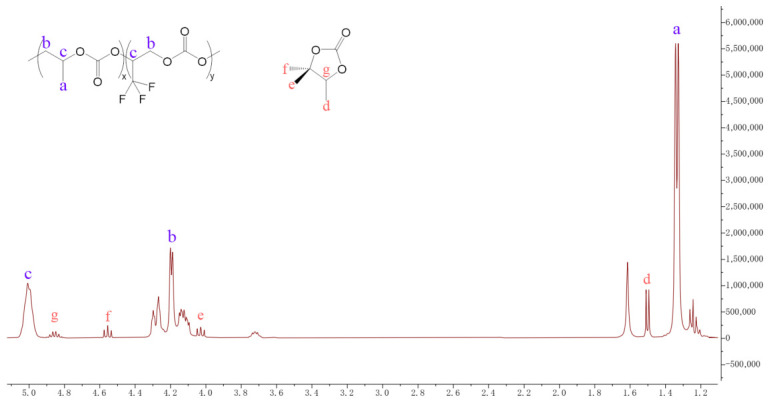
^1^H NMR spectrum of PPCF.

The ^1^H NMR spectrum of PPCF (Figure 6) displays several resolved signals that can be assigned as follows. The resonance at 1.33 ppm (labeled **a**) is attributed to the methyl protons of the ring-opened propylene oxide (PO) units. The signal at 1.51 ppm (**d**) corresponds to the methyl protons of a minor propylene carbonate cyclic byproduct. The broad signal envelope observed between 4.1 and 4.3 ppm (**b**) encompasses the methylene (-CH_2_-) protons of both PO and TF-PO backbone units. The considerable broadening and overlapping of signals in this region can be rationalized by the DFT-optimized geometry, which reveals a distribution of local chain conformations and varying chemical environments for these methylene protons, leading to a quasi-continuous distribution of chemical shifts that coalesce into the observed broad feature. Additional resolved contributions from the cyclic carbonate byproduct appear as distinct methylene resonances at 4.03 ppm (**e**) and 4.56 ppm (**f**), while its methine proton is observed at 4.85 ppm (**g**). The downfield signal at 5.01 ppm (**c**) integrates the methine protons of the main-chain PO unit and the -CH(CF_3_)^-^ proton of the incorporated TF-PO unit.

Quantitative analysis based on signal integration provides insight into the copolymer composition. When the integral of signal **c** (methine protons, 5.01 ppm) is normalized to 1.00, representing one molar equivalent of combined PO and TF-PO repeat units, the integral of signal **b** (methylene protons, 4.1–4.3 ppm) is found to be 2.02, which is in close agreement with the expected value of 2.00 for two protons per repeat unit. Concurrently, the methyl resonance of the PO unit (**a**, 1.33 ppm) integrates to 2.87. From the relative intensity of signal **a** (2.87 out of an expected 3.00 protons per PO unit), the molar fraction of PO in the copolymer chain is estimated as 2.87/3≈0.957, with the remaining fraction of 1−0.957=0.043 attributed to TF-PO units. This corresponds to a PO:TF-PO molar ratio of approximately 22:1 in the copolymer. Compared with the initial feed ratio of 100:4 (i.e., 25:1), this result indicates that TF-PO is incorporated into the polymer backbone with a slight preference relative to PO, consistent with its higher reactivity under the applied catalytic conditions. In related CO_2_/epoxide copolymerization systems, Wang and Mu reported that under optimized conditions the carbonate linkage content can exceed 99% with minimal ether linkage formation [53].

### 3.2. Thermal Property Characterization of PPCF

**Figure 7:**
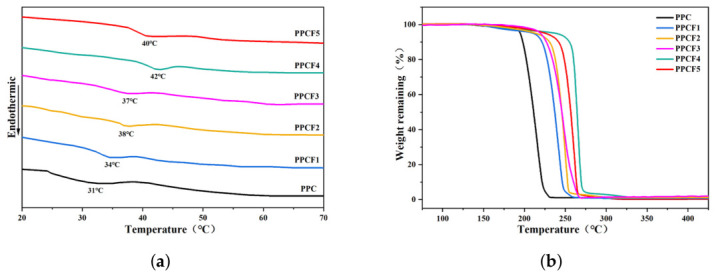
(**a**) DSC curves of PPC and PPCFs; (**b**) TGA curves of PPC and PPCFs.

Thermal performance analysis was conducted using differential scanning calorimetry (DSC) and thermogravimetric analysis (TGA) (Figure 7). DSC results revealed that all polymers exhibited amorphous structures with a single glass transition temperature ($T_{g}$). After the introduction of TF-PO, the $T_{g}$ of PPCF significantly increased, reaching up to 42 °C—an 11 °C improvement over PPC—and could be regulated by varying the monomer feed ratio (Table 8). The elevation in $T_{g}$ is directly associated with the restricted segmental mobility of the polymer chains [13]. The incorporation of the bulky and highly polar -CF_3_ side groups from TF-PO imposes topological constraints and enhances interchain dipole–dipole interactions, thereby impeding the large-scale conformational motions responsible for the glass transition.

TGA demonstrated a notable enhancement in thermal stability after modification: the 5% weight loss temperature ($T_{d,-5\%}$) of PPCF4 was 242 °C (an increase of 47 °C), while the maximum decomposition temperature ($T_{d,max}$) reached 272 °C (an increase of 61 °C). This substantial improvement in thermal resistance can be rationalized by two primary mechanisms. Firstly, the strong electron-withdrawing nature and high bond energy of the C-F bonds increase the overall thermal stability of the backbone. More importantly, the thermal degradation of unmodified PPC is known to proceed predominantly via an “unzipping” mechanism, wherein the terminal hydroxyl groups act as initiation sites for chain scission [13,25]. The introduction of TF-PO units along the copolymer chain effectively dilutes the concentration of these vulnerable chain ends and creates steric barriers that hinder the propagation of the unzipping reaction [25]. Furthermore, the rigid nature of the fluorinated segments restricts the conformational flexibility required for the cyclic depolymerization pathway, thereby enhancing the overall thermal endurance of the PPCF materials.

### 3.3. Mechanical Property Characterization of PPCF

**Figure 8:**
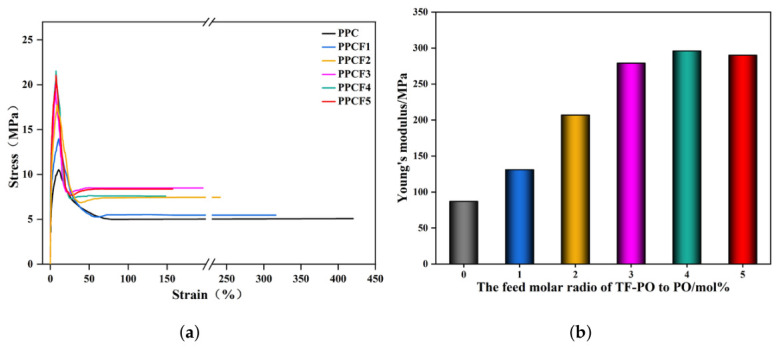
(**a**) Stress–strain curves of PPC and PPCFs; (**b**) Young’s modulus of PPC and PPCFs.

To address the insufficient mechanical properties of poly(propylene carbonate) (PPC) caused by facile chain slippage, this study introduced 1,1,1-trifluoro-2,3-epoxypropane (TF-PO) containing C-F bonds for structural modification. The results (Figure 8, Table 9) demonstrate that the modified PPCFs exhibited significant improvements in tensile strength and Young’s modulus. Specifically, PPCF4 achieved a tensile strength of 21.5 MPa and a Young modulus of 296 MPa, representing increases of 106.7% and 240%, respectively, compared to pristine PPC. Concurrently, the elongation at break decreased to a minimum of 148%, corresponding to a reduction of 64.8%.

The remarkable enhancement in mechanical stiffness and strength can be attributed to the intrinsically rigid and highly polar character of the fluorinated segments [25]. The strong electron-withdrawing nature of the -CF_3_ group intensifies the polarity of the polymer backbone, thereby augmenting intermolecular dipole–dipole interactions and cohesive energy density [15]. This reinforcement of secondary bonding forces effectively restricts the relative slippage of polymer chains under external stress. Moreover, analogous to the reinforcement mechanism observed in PPC blends with rigid moieties, the incorporation of TF-PO restricts the segmental mobility of the polymer chains, which not only elevates the glass transition temperature but also translates directly into enhanced elastic modulus and tensile resistance [15,25]. The observed decrease in elongation at break is a typical trade-off in rigidified polymer systems, wherein the diminished chain flexibility and restricted plastic deformation lead to a more brittle fracture mode compared to the ductile nature of unmodified PPC [16]. Consequently, the strategic introduction of fluorinated comonomer units enables a tailored balance between stiffness and ductility, effectively expanding the potential for industrial applications of PPC-based materials.

### 3.4. Surface Property Characterization of PPCF

**Figure 9:**
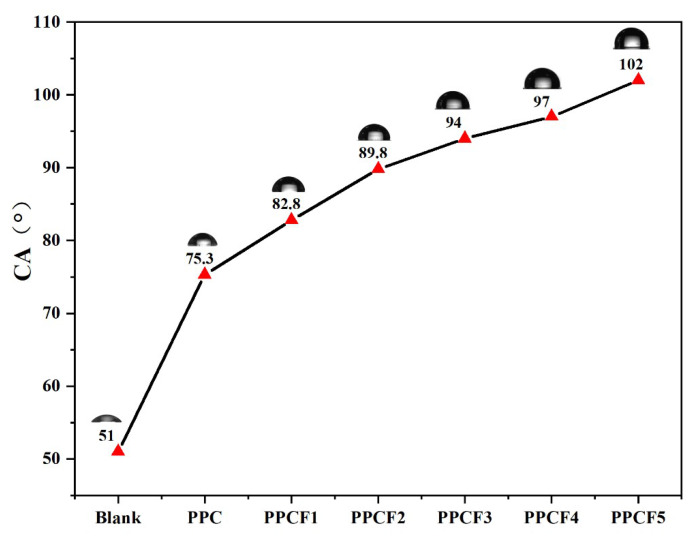
Water contact angles of the PPCF copolymer series.

Contact angle measurements were employed to evaluate the hydrophobicity of the materials (Figure 9). The results show that the contact angle of a blank glass slide was 51°, indicating hydrophilic characteristics. As the feed ratio of TF-PO increased from 0% to 3%, the contact angle increased from 75.3° to 94°, indicating a transition from hydrophilic to hydrophobic properties. A further increase to 5% raised the contact angle to 102°. The initial hydrophobicity of pristine PPC originates from its methyl side groups, while the subsequent significant improvement is attributed to the introduction of C-F bonds. It is well documented that -CF_3_ groups possess an intrinsically low surface energy (approximately 6 mN/m) compared to -CH_3_ groups (22-24 mN/m) owing to the low polarizability and weak intermolecular interactions of fluorinated moieties [54]. This reduction in surface energy effectively hinders hydrogen bond formation with water molecules, thereby imparting the material with enhanced hydrophobic characteristics. Furthermore, the self-segregation and enrichment of fluorinated chain segments at the air–polymer interface is a common driving force for achieving low surface energy in fluorinated coatings [55]. The XPS results (Figure 4 and Table 7) corroborate this phenomenon, revealing a substantial surface fluorine content that exceeds the bulk stoichiometric ratio, which accounts for the observed macroscopic hydrophobicity.

**Figure 10:**
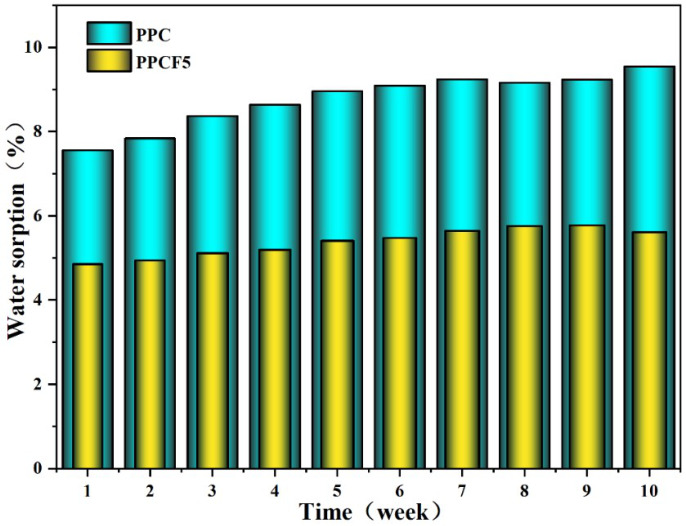
Variation in water absorption rates of PPC and PPCF5 over time in Type III water.

Water absorption rate is a key indicator for evaluating the waterproof performance of materials. The test was conducted according to the Chinese standard JC/T 2663-2022 [56] at (23±2) °C and (50±5)% relative humidity. Consistent with the contact angle test results, the modified PPCF5 exhibited excellent waterproof characteristics. As shown in Figure 10, after 70 days of immersion, the water absorption rate of PPCF5 stabilized at approximately 5.4%, with no significant discoloration, cracking, or swelling observed in the material. In contrast, the water absorption rate of unmodified PPC increased from 7.6% to 9.5%, which may be attributed to the cleavage of ester groups in its molecular chains generating hydrophilic hydroxyl groups, thereby promoting water absorption and material degradation. These results demonstrate that the introduction of the C-F-containing monomer significantly enhances the material’s hydrophobicity and durability.

**Figure 11:**
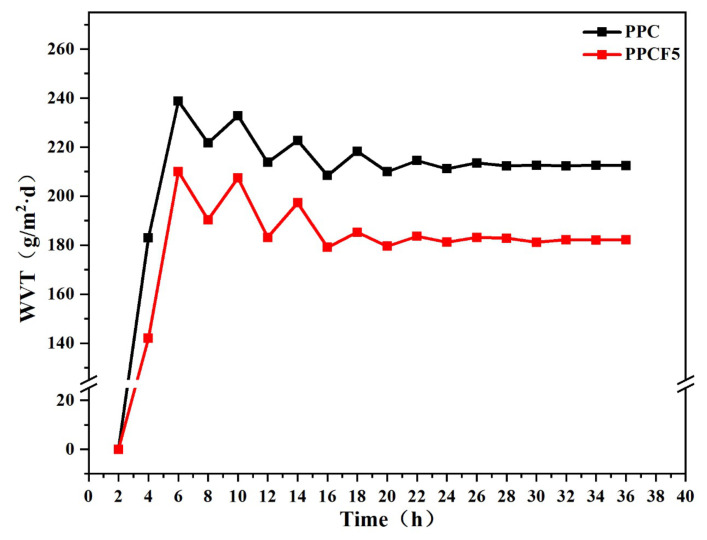
Water vapor transmission rates of PPC and PPCF5 films over time.

To evaluate the moisture barrier properties of the materials, water vapor transmission (WVT) tests were conducted over 36 h on PPC and PPCF5 films according to the Chinese standard GB/T2021 [57] at (23±2) °C and (90±2)% relative humidity (Figure 11). The results show that the WVT of PPCF5 eventually stabilized at $165.2\;{g/m}^{2}\cdot d$, significantly lower than the $212.4\;{g/m}^{2}\cdot d$ of unmodified PPC. This improvement is attributed to the introduction of hydrophobic C-F bonds, which reduce direct contact between water and the polymer surface. Additionally, the fluorinated segments tend to form a densely packed and ordered shell-like structure at the surface, as observed in analogous fluorinated block copolymer systems [58], which acts as an effective barrier against the permeation of water vapor molecules. The synergistic effect of reduced surface energy and a more compact interfacial layer leads to the observed enhancement in water vapor barrier performance.

### 3.5. Dielectric Property Characterization of PPCF

**Figure 12:**
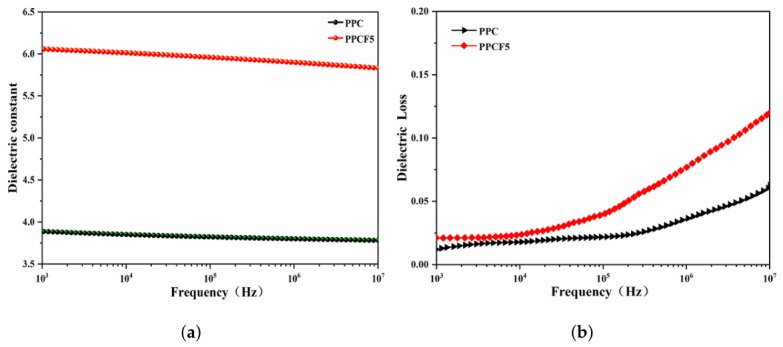
(**a**) Dielectric constant curves; (**b**) Dielectric loss curves of PPC and PPCF5.

As shown in Figure 12, the incorporation of TF-PO units (approximately 4.3 mol% as quantified by ^1^H NMR, corresponding to a PO:TF-PO molar ratio of ∼22:1 in the copolymer, Figure 6) significantly enhances the dielectric properties of PPCF5. The dielectric constant increases from 3.85 (PPC) to 6.01 at $10^{4}$ Hz and remains stable across $10^{3}$–$10^{7}$ Hz (Figure 12a), satisfying the requirement for solid electrolytes (ε≥5). This improvement is attributed to the strong dipole moment of the introduced C-F bonds, which increase the overall polarizability of the polymer chains. A similar enhancement in dielectric constant through the introduction of highly polar side groups has been reported in sulfone-containing polycarbonate copolymers, where the increased dipole polarization arising from the electron-withdrawing moieties directly elevates the dielectric response [59]. Furthermore, it is well documented that -CF_3_ groups possess an intrinsically strong dipole moment, which facilitates orientational polarization under an external electric field and contributes substantially to the observed increase in dielectric constant [60].

Regarding dielectric loss (Figure 12b), PPCF5 exhibits consistently higher tanδ values than PPC across the entire frequency range, with the disparity progressively widening at higher frequencies. This behavior reflects the combined effects of two factors: first, the enhanced dipolar polarization from the introduced C-F moieties contributes to greater energy dissipation during dipole reorientation, and second, the strongly electron-withdrawing nature of the CF_3_ groups intensifies the local polarity and enhances interchain dipole-dipole interactions, which elevates the glass transition temperature (from 31 to 42 °C) and modifies the dynamic response of the polymer chains to the alternating electric field. At higher frequencies, where dipole orientation becomes increasingly hindered, the more polar PPCF5 chains exhibit greater resistance to segmental motion, resulting in higher energy dissipation compared to the less polar PPC chains. This interpretation is consistent with broadband dielectric relaxation studies on polycarbonate, which demonstrate that the α-relaxation process associated with the glass transition is highly sensitive to chain rigidity and intermolecular constraints; an increase in $T_{g}$ shifts the relaxation spectrum to lower frequencies and alters the temperature- and frequency-dependence of the dielectric loss [61]. These results demonstrate that controlled incorporation of TF-PO units enables systematic tuning of both dielectric constant and loss behavior through the combined effects of enhanced polarizability and modified chain dynamics imparted by the highly polar C-F and CF_3_ groups.

## 4. Discussion

### 4.1. Computational Results and Analysis

**Figure 13:**
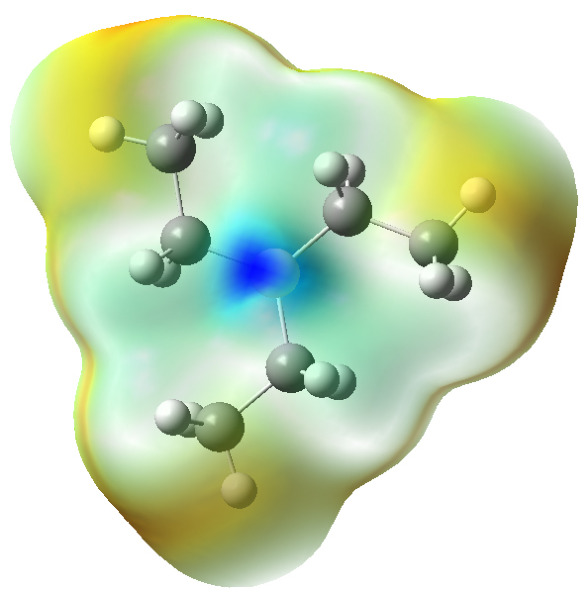
Electrostatic potential distribution of triethylborane (TEB). In this figure, areas that appear more blue indicate stronger positive charge distribution.

**Figure 14:**
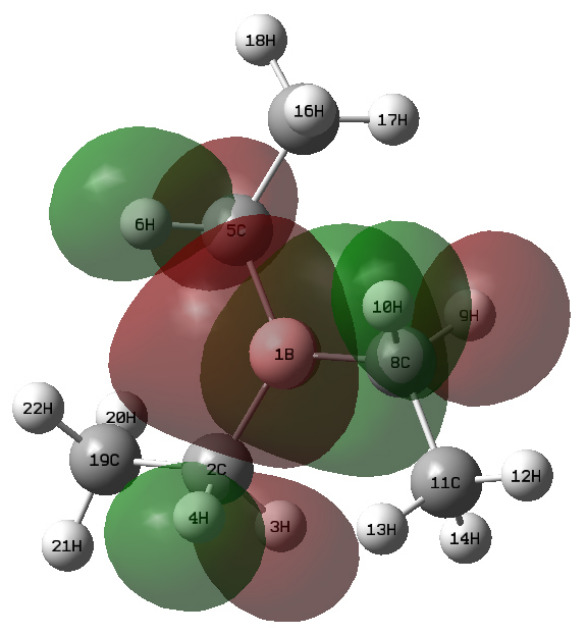
Geometry of the lowest unoccupied molecular orbital (LUMO) of triethylborane (TEB).

In triethylborane (TEB), the carbon atoms are more electronegative than boron, resulting in a distinct positive charge around the boron atom (Figure 13). The boron atom is sp^2^-hybridized, adopting a trigonal planar geometry, with the three sp^2^ hybrid orbitals involved in bonding and one vacant p orbital perpendicular to the molecular plane. This unoccupied orbital enhances the electron-deficient nature of boron, rendering it Lewis acidic. Computational results indicate that the boron atom contributes predominantly to the lowest unoccupied molecular orbital (LUMO) (Table 10), confirming that the LUMO is primarily localized on the empty p orbital of boron, which constitutes the Lewis acidic active site (as shown in Figure 14).

**Figure 15:**
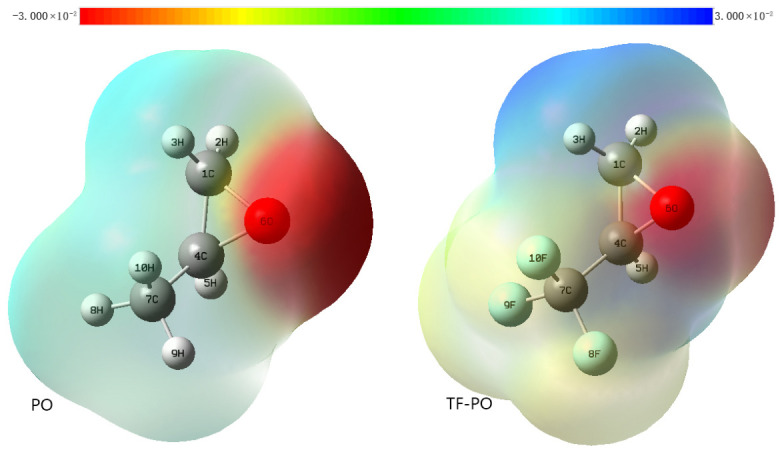
Electrostatic potential distribution. **Left**: PO; **Right**: TF-PO.

**Figure 16:**
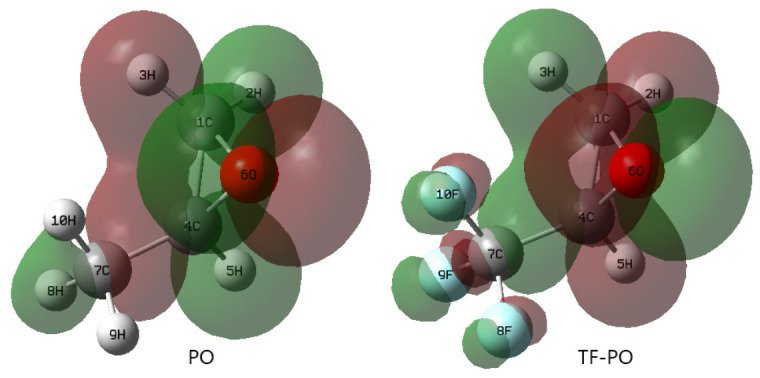
Highest occupied molecular orbital (HOMO). **Left**: PO; **Right**: TF-PO.

**Figure 17:**
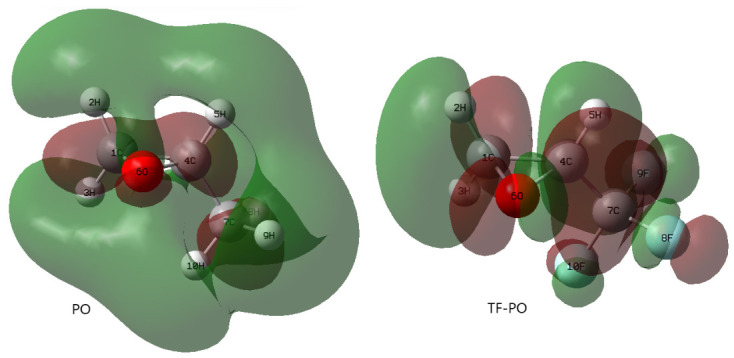
Lowest unoccupied molecular orbital (LUMO). **Left**: PO; **Right**: TF-PO.

Propylene oxide (PO) and 1,1,1-trifluoro-2,3-epoxypropane (TF-PO) share certain similarities:

(1) Examination of their charge distributions (Figure 15) reveals that the negative charge is primarily localized on the oxygen atoms in both molecules. When interacting with a Lewis acid such as TEB, the highest occupied molecular orbital (HOMO) is contributed predominantly by the oxygen atoms (Figure 16, Table 11 and Table 12). This indicates that both PO and TF-PO readily act as Lewis bases through their oxygen atoms, providing electron pairs.

(2) Analysis of the LUMO of PO and TF-PO (Figure 17) reveals that the lowest unoccupied molecular orbital of PO is primarily contributed by carbon atoms 1, 4, and 7 (Table 13), while that of TF-PO is mainly contributed by carbon atoms 1 and 4 (Table 14). These carbon atoms can serve as Lewis acidic sites. The Cl^−^ ion, acting as a nucleophile, facilitates the release of ring strain by attacking the carbon atom with higher positive charge in the epoxide ring, thereby activating the ring-opening process (the atom numbering in the text corresponds to that in Figure 15, Figure 16 and Figure 17).

DFT calculations also provide compelling evidence for the higher reactivity of TF-PO compared to PO in the TEB/PPNCl-catalyzed terpolymerization. The LUMO energy of TF-PO is calculated to be 0.06632 Hartree, significantly lower than that of PO (0.10578 Hartree). This lower LUMO energy facilitates nucleophilic attack by the Cl^−^ ion, thereby reducing the energy barrier for ring-opening and enhancing the reactivity of TF-PO. Furthermore, frontier molecular orbital analysis reveals that the LUMO of TF-PO is primarily localized on the epoxide ring carbons, making it more susceptible to ring-opening. Additionally, the dipole moment of TF-PO (2.3442 D) is larger than that of PO (1.9707 D), indicating stronger electrostatic interactions with the Lewis acidic boron center of TEB, which further promotes its activation. The HOMO-LUMO energy gap of TF-PO (0.36187 Hartree) is also slightly narrower than that of PO (0.36823 Hartree), suggesting an overall higher reactivity. These computational results demonstrate that TF-PO possesses intrinsically higher reactivity than PO under the employed catalytic conditions, confirming its ability to actively participate in the ternary copolymerization.

These computational findings are in excellent agreement with the quantitative ^1^H NMR analysis of the resulting copolymer. As shown in Figure 6, the integrated signal intensities reveal a PO:TF-PO molar ratio of approximately 22:1 in the copolymer, whereas the initial feed ratio was 100:4 (i.e., 25:1). The slightly higher incorporation of TF-PO relative to the feed composition can be directly attributed to its intrinsically higher reactivity, as predicted by the lower LUMO energy and larger dipole moment. Consequently, the DFT results not only rationalize the preferential insertion of TF-PO into the growing polymer chain but also substantiate the quantitative NMR findings, confirming that TF-PO actively participates in the ternary copolymerization under the employed catalytic conditions.

### 4.2. Proposed Mechanism for the Ternary Copolymerization

**Figure 18:**
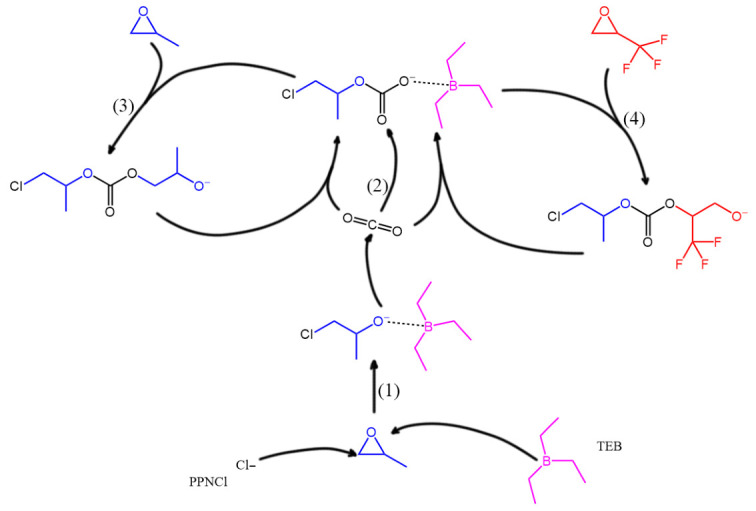
Proposed mechanism for the ternary copolymerization catalyzed by the metal-free TEB/PPNCl system.

Based on the DFT calculations and experimental observations, a plausible mechanism for the TEB/PPNCl-catalyzed ternary copolymerization of CO_2_, PO, and TF-PO is proposed, as illustrated in Figure 18. The catalytic cycle proceeds through a sequence of elementary steps that involve cooperative Lewis acid–base interactions and alternating monomer insertions.

**Figure 19:**
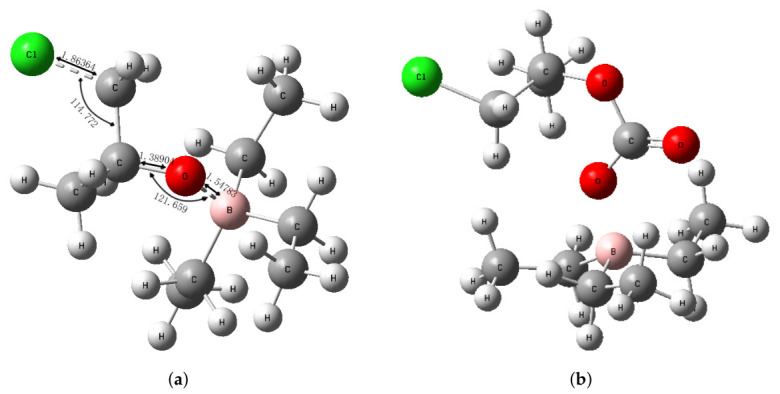
Optimized structures of key intermediates in the TEB/PPNCl-catalyzed copolymerization: (**a**) alkoxide anion species resulting from concerted ring-opening of PO; (**b**) carbonate-terminated chain end after CO_2_ insertion.

**(1) Epoxide Ring-Opening Initiation.** The Lewis acidic boron center of TEB coordinates to the oxygen atom of PO, polarizing the epoxide C-O bond and rendering the adjacent methylene carbon more electrophilic. Concurrently, the nucleophilic chloride anion (Cl^−^) from PPNCl attacks the less substituted methylene carbon of the coordinated PO. This concerted activation facilitates the heterolytic ring-opening of the epoxide, generating a ring-opened alkoxide intermediate. In this transition state (Figure 19a), the negative charge originally localized on the chloride ion is transferred to the oxygen atom of the cleaved epoxide, yielding an alkoxyborane species stabilized by the B-O interaction.

**(2) Carbon Dioxide Insertion.** The nucleophilic oxygen atom of the alkoxide intermediate, bearing partial negative charge, attacks the electrophilic carbon atom of a CO_2_ molecule. This insertion step results in the formation of a carbonate-terminated chain end, wherein the negative charge is now delocalized onto one of the oxygen atoms of the newly incorporated carbonate group (Figure 19b). The resulting linear carbonate intermediate serves as the active species for subsequent epoxide ring-opening events.

**(3) Propylene Oxide Propagation.** The negatively charged oxygen atom of the carbonate intermediate, assisted by the Lewis acidic TEB, nucleophilically attacks a new PO monomer. This step mirrors the initiation process and leads to the regeneration of an alkoxide chain end while extending the polymer backbone by one propylene carbonate unit. The alternation between CO_2_ insertion (step 2) and PO ring-opening (step 3) constitutes the principal propagation pathway for the formation of PPC segments.

**(4) 1,1,1-Trifluoro-2,3-epoxypropane (TF-PO) Incorporation.** In a competing and alternating fashion, the carbonate-terminated active chain end can also attack a TF-PO monomer, again with assistance from TEB. Due to the electron-withdrawing nature of the trifluoromethyl (-CF_3_) group, TF-PO exhibits a lower LUMO energy and a larger dipole moment compared to PO, as revealed by DFT calculations, rendering it more susceptible to nucleophilic attack. This step results in the insertion of a fluorinated propylene carbonate unit into the polymer chain, thereby introducing the functional -CF_3_ side groups.

The polymerization proceeds through repetitive cycles of CO_2_ insertion (step 2) followed by either PO ring-opening (step 3) or TF-PO ring-opening (step 4), thereby yielding the alternating fluorinated poly(propylene carbonate) (PPCF) copolymer. The dual role of TEB—activating both the epoxide monomer and the CO_2_ molecule while stabilizing the propagating alkoxide intermediate—is essential for achieving high catalytic activity and selectivity toward carbonate linkages over ether linkages.

## 5. Conclusions

In this study, a series of fluorinated poly(propylene carbonate) (PPCF) materials were successfully synthesized via the metal-free TEB/PPNCl-catalyzed terpolymerization of CO_2_, propylene oxide, and 1,1,1-trifluoro-2,3-epoxypropane (TF-PO). The optimal polymerization conditions were established, yielding copolymers with controlled molecular weight and narrow polydispersity. Structural characterization confirmed the successful incorporation of TF-PO units into the polymer backbone, and quantitative ^1^H NMR analysis revealed an enrichment of the fluorinated monomer relative to the feed, consistent with DFT predictions of its higher intrinsic reactivity.

The introduction of fluorinated side groups led to substantial improvements in material performance. Compared to unmodified PPC, the PPCF copolymers exhibited significantly enhanced thermal stability, elevated glass transition temperature, and markedly improved tensile strength and Young’s modulus. Furthermore, the materials demonstrated pronounced hydrophobicity with increased water contact angles and reduced water vapor transmission, as well as a notable enhancement in dielectric constant owing to the strong dipole polarization of the C–F bonds.

DFT calculations elucidated the catalytic mechanism, confirming the Lewis acidic role of TEB and rationalizing the preferential incorporation of TF-PO based on frontier orbital analysis. This work demonstrates that the strategic introduction of a fluorinated comonomer is an effective molecular design approach to overcome the intrinsic limitations of PPC, yielding sustainable materials with a desirable combination of thermal, mechanical, hydrophobic, and dielectric properties for potential applications in advanced packaging and electronic devices.

## Figures and Tables

**Table 1:** Binary interaction parameter $k_{ij}$ for the CO_2_ (1)–propylene oxide (2) system.

$k_{\mathrm{ij}}$	CO_2_	PO
CO_2_	0.0000	−0.0057
PO	−0.0057	0.0000

**Table 2:** Pure-component critical properties and acentric factors used in the SRK equation of state [51].

Component	$T_{c}$ (K)	$P_{c}$ (bar)	ω
CO_2_	304.18	73.82	0.2255
Propylene oxide (PO)	482.31	49.12	0.2579

**Table 3:** Effect of Reaction Temperature on Copolymerization Results ^*a*^.

Temperature (°C)	Yield (%)	${\bar{M}}_{n}$ (kDa)	PDI
40	17	4.21	2.43
50	55	9.43	1.76
60	78	20.67	1.18
70	71	11.45	1.49

^*a*^ Reaction conditions: TEB:PPNCl = 2:1, CO_2_ pressure = 2 MPa, reaction time = 12 h.

**Table 4:** Effect of Reaction Pressure on Copolymerization Results ^*a*^.

Pressure (MPa)	Yield (%)	${\bar{M}}_{n}$ (kDa)	PDI
0.5	9	2.16	2.93
1	58	9.74	1.85
2	78	20.67	1.18
3	73	16.45	1.32

^*a*^ Reaction conditions: TEB:PPNCl = 2:1, reaction temperature = 60 °C, reaction time = 12 h.

**Table 5:** Effect of Reaction Time on Copolymerization Results ^*a*^.

Time (h)	Yield (%)	${\bar{M}}_{n}$ (kDa)	PDI
4	25	4.87	1.92
8	61	10.36	1.55
12	78	20.67	1.18
16	80	19.11	1.37

^*a*^ Reaction conditions: TEB:PPNCl = 2:1, reaction temperature = 60 °C, reaction pressure = 2 MPa.

**Table 6:** Polymerization results of PPCF products ^*a*^.

Copolymer	Molar Ratio n[PO]:n[TF-PO]	Yield (%)	${\bar{M}}_{n}$ (kDa)	PDI
PPC	1:0	68	8.10	1.23
PPCF1	100:1	45	9.23	1.22
PPCF2	100:2	56	10.16	1.20
PPCF3	100:3	78	20.67	1.18
PPCF4	100:4	81	21.25	1.13
PPCF5	100:5	80	20.61	1.15

^*a*^ Reaction conditions: TEB:PPNCl = 2:1, reaction temperature = 60 °C, reaction pressure = 2 MPa, reaction time = 12 h.

**Table 7:** Surface elemental composition of PPC and PPCF determined by XPS.

Sample	C (at. %)	O (at. %)	F (at. %)
PPC	61.2	38.8	–
PPCF	45.9	17.2	36.9

**Table 8:** Thermal Properties of PPC and PPCFs.

Copolymer	$T_{g}$ (°C)	$T_{d,-5\%}$ (°C)	$T_{d,\max}$ (°C)
PPC	31	195	211
PPCF1	34	213	249
PPCF2	38	215	254
PPCF3	37	220	247
PPCF4	42	242	272
PPCF5	40	226	266

**Table 9:** Mechanical properties of PPC and PPCFs.

Copolymer	Tensile Strength (MPa)	Elongation at Break (%)	Young’s Modulus (MPa)
PPC	10.4	420	87
PPCF1	14.1	316	131
PPCF2	17.2	241	207
PPCF3	19.6	196	279
PPCF4	21.5	148	296
PPCF5	20.3	157	290

**Table 10:** Contribution coefficients of atomic orbitals to the lowest unoccupied molecular orbital (LUMO) of TEB.

Atomic Orbital	2P_X_	2P_Y_	2P_Z_	3S	3P_X_	3P_Y_	3P_Z_
**B** Contribution Coefficient	−0.00002	−0.00001	0.49852	−0.00023	−0.00003	0.00000	0.63354

**Table 11:** Contribution coefficients of atomic orbitals to the highest occupied molecular orbital (HOMO) of PO.

Atomic Orbital	2P_X_	2P_Y_	2P_Z_	3S	3P_X_	3P_Y_	3P_Z_
**O** Contribution Coefficient	−0.31212	−0.13133	0.49873	−0.01757	−0.24266	−0.10179	0.36811

**Table 12:** Contribution coefficients of atomic orbitals to the highest occupied molecular orbital (HOMO) of TF-PO.

Atomic Orbital	2P_X_	2P_Y_	2P_Z_	3S	3P_X_	3P_Y_	3P_Z_
**O** Contribution Coefficient	0.32184	−0.09541	0.49832	0.04095	0.25594	−0.07513	0.35789

**Table 13:** Contribution coefficients of atomic orbitals to the lowest unoccupied molecular orbital (LUMO) of PO.

Atomic Orbital	1S	2S	2P_X_	2P_Y_	2P_Z_	3S	3P_X_	3P_Y_	3P_Z_
1**C** Contribution	−0.07629	0.14273	−0.09850	0.07572	−0.03371	1.01563	−0.38372	0.26730	−0.21163
4**C** Contribution	−0.05431	0.09902	−0.01481	−0.04348	0.12227	0.88626	−0.10601	−0.11817	0.34376
7**C** Contribution	−0.08539	0.10504	0.13579	0.01426	−0.08109	1.41883	0.40650	−0.01444	−0.31563

**Table 14:** Contribution coefficients of atomic orbitals to the lowest unoccupied molecular orbital (LUMO) of TF-PO.

Atomic Orbital	1S	2S	2P_X_	2P_Y_	2P_Z_	3S	3P_X_	3P_Y_	3P_Z_
1**C** Contribution	−0.04819	0.06758	0.23633	−0.16302	−0.18614	0.69451	0.40679	−0.19192	−0.35320
4**C** Contribution	0.00855	0.01087	−0.01781	0.36456	0.06483	−0.29958	0.05155	0.67229	0.06424
7**C** Contribution	−0.00330	0.00899	−0.02479	0.27833	0.03787	0.04042	−0.02447	0.21573	0.03125

## Data Availability

The original contributions presented in this study are included in the article. Further inquiries can be directed to the corresponding author.

## Abbreviations

The following abbreviations are used in this manuscript:

PPC	Poly(propylene carbonate)
PPCF	Fluorinated poly(propylene carbonate)
TF-PO	1,1,1-Trifluoro-2,3-epoxypropane
PO	Propylene oxide
TEB	Triethylborane
PPNCl	Bis(triphenylphosphine)iminium chloride
